# Oral language comprehension interventions in 1–8-year-old children with language disorders or difficulties: A systematic scoping review

**DOI:** 10.1177/2396941520946999

**Published:** 2020-08-20

**Authors:** Sirpa Tarvainen, Suvi Stolt, Kaisa Launonen

**Affiliations:** Department of Psychology and Logopedics, Unit of Logopedics, University of Helsinki, Helsinki, Finland; Department of Psychology and Logopedics, Unit of Logopedics, University of Helsinki, Helsinki, Finland

**Keywords:** Specific language impairment, speech and language therapy, focus of intervention, efficacy, level of evidence

## Abstract

**Background and aims:**

The most severe problems in language manifest as difficulties in comprehending oral language. These difficulties are persistent and expose individuals to several risk factors. There is a lack of intervention research in the area of oral language comprehension, and no reviews have focused solely on oral language comprehension interventions in young children. The aim of this review was to identify interventions targeting oral language comprehension in children 8 years or younger with language disorders or difficulties. The review also examined the possible intervention foci, efficacy, and level of evidence of these interventions.

**Methods:**

A systematic scoping review of eight databases was carried out. Twenty of 2399 articles met the inclusion criteria and a further six articles were identified through reference lists of sourced articles. These 26 articles described 25 studies. Altogether 2460 children aged 1–8 years participated in the 25 studies. The data from these studies were extracted and analysed, and the intervention foci, efficacy, and level of evidence were evaluated.

**Main contribution:** The reviewed interventions focused on three aspects: modifying the communicative environment of the child; targeting aspects of the child’s language; or targeting the child’s language processing. Of the included studies, 80% indicated positive effects on participants’ oral language comprehension. The level of evidence of the included studies varied. With few exceptions, researchers and practitioners can have moderate confidence in the results of the included studies indicating that it is possible to ameliorate difficulties in oral language comprehension.

**Conclusions:**

This review summarises the existing evidence on oral language comprehension interventions in young children with language disorders or difficulties. The evidence base is still limited, and more research is urgently needed. The results suggest that though not all interventions seem to provide desired outcomes, there are several interventions indicating efficacy to target problems in oral language comprehension in 1–8-year-old children with language disorders or difficulties. A careful choice of therapy technique and collaboration with people in the child’s environment is required to maximize outcomes.

**Implications:** The results suggest that young children’s oral language comprehension skills can be improved by guiding parents and clinicians in their communication strategies, and by clinician-implemented interventions targeting aspects of the child’s language. The research on interventions targeting children’s language processing is limited, and the results mixed. The present study provides information on different oral language comprehension interventions and their outcomes. The findings are readily applicable for clinical use.

## Introduction

Difficulties in oral language comprehension refer to problems in comprehending spoken language. The term ‘receptive language’ is also used for comprehension of oral language.^1^ One of the most common causes of receptive language difficulties is developmental language disorder (DLD). It refers to language difficulties, diagnosed in the absence of any obvious cause, that affect functional communication in everyday life and have a poor prognosis (Bishop et al., 2017). A large discrepancy between nonverbal and verbal ability is not required for a diagnosis of DLD. The term DLD has recently been proposed to replace previously used terms (Bishop et al., 2017), such as specific language impairment (SLI) and language impairment (LI). Reported DLD prevalence rates vary from 7% to 19% (McKean et al., 2017; Norbury et al., 2016; Tomblin et al., 1997). Children with DLD can have difficulties in expressive and/or receptive language. The most severe language disorders manifest as problems in comprehending oral language (Saar et al., 2018). Children that experience difficulties in oral language comprehension have been recognised as needing long-term support (Clark et al., 2007).

Difficulties in oral language comprehension can also occur alongside other diagnoses, for example, language disorders may be associated with biomedical conditions, such as Down syndrome or autism spectrum disorders (Bishop et al., 2017). Children can have difficulties in comprehending oral language also due to other reasons. For example, low socio-economic status is associated with poor language skills (Fernald et al., 2013). Children with less severe difficulties do not necessarily qualify for a diagnosis, although they may still require support in language and learning (Bishop et al., 2017).

Prediction of outcomes and diagnosing DLD is particularly difficult in children under three years-of-age (Bishop et al., 2017). Young children thus rarely receive a diagnosis of DLD. Further, as stated above, the origin of difficulties in oral language comprehension can vary. Therefore, in the present review, the concept ‘children with language disorders or difficulties’ is used to refer to children with varying language difficulties.

The trajectories of children with language disorders differ according to age (Bishop et al., 2017). Difficulties are likely to persist in children that are 5 years and older (Stothard et al., 1998). In particular difficulties in oral language comprehension predict persistent language difficulties (Bishop et al., 2017; O’Neill et al., 2019; Roberts & Kaiser, 2012). Children with difficulties in oral language comprehension are thus at a greater risk for persistent language difficulties than children with difficulties in expressive language only. Longitudinal studies indicate that persistent language difficulties expose individuals to a number of risks, including poor social relations (Durkin & Conti-Ramsden, 2007), depression and anxiety (Botting et al., 2016), low literacy, unemployment, and low socioeconomic status (Elbro et al., 2011), and juvenile criminality (Bryan et al., 2007). These risks could be minimised, and difficulties ameliorated, if children with language comprehension difficulties received effective interventions. Speech and language therapy interventions have the potential to enhance the quality of life of the individual with language difficulties, and to diminish societal costs (Marsh et al., 2010).

### Focus of intervention

Several skills are needed to comprehend oral language: speech processing at an auditory and sound level, knowing the meaning of words, understanding the grammatical structures that words form, retaining all this information while completing the previously mentioned tasks, and integrating it within the context in which it is said (Morgan, 2013). Oral language comprehension interventions can target one or more of these skills. The focus of an intervention is the skill, area, or feature that is targeted. The following areas have been mentioned as possible foci of oral language comprehension intervention: (1) auditory processing; (2) language processing; (3) receptive syntax; (4) receptive morphology; (5) receptive vocabulary; (6) semantics; (7) narratives, and (8) both expressive and receptive language together (Boyle et al., 2010; Cirrin & Gillam, 2008; Law et al., 2003, 2004). Areas 1–2, auditory and language processing, refer to processes influencing language skills in general. The underlying idea of interventions targeting language processing is that language skills improve as a result of improved processing skills. Areas 3–8 refer to aspects of the child’s language. The provider of an intervention has usually been a clinician, typically a speech and language therapist (Cirrin & Gillam, 2008; Law et al., 2003).

The International Classification of Functioning, Disability and Health (ICF) (World Health Organization, 2013) provides a classification of health-related domains. When we compare the intervention foci of previous research to the ICF framework, it is clear that the interventions have focused only on the health condition: activity limitations have been targeted by improving language or language-processing skills. For example, language difficulties have been ameliorated by improving receptive morphology (Cirrin & Gillam, 2008). However, the ICF framework also emphasises the role of contextual factors, such as environmental factors. These environmental factors, including communicative environment, have received little attention in oral language comprehension interventions. Language comprehension interventions can also target parents’ communication skills (Roberts & Kaiser, 2011). Therefore, the intervention foci identified in previous reviews may not be sufficient, and another kind of classification of intervention foci may be justified. Given the complexity of oral language comprehension, it is important that we deepen our understanding of the typology of these interventions. This understanding of the intervention typology may lead to more possible avenues for interventions being considered.

### Efficacy of oral language comprehension interventions

Evidence for the efficacy of oral language comprehension interventions is contradictory and sparse. In the present review efficacy refers to the degree of ability to produce a desired effect. Effect size is considered to express the magnitude of efficacy. To our knowledge, there are no systematic reviews or meta-analyses focusing solely on the efficacy of oral language comprehension interventions. Law et al. (2003, 2004) published a meta-analysis of speech and language therapy interventions, including oral language comprehension interventions. In this study, the efficacy of speech and language therapy interventions for children and adolescents (0–15 years) with primary speech and language delay or disorder was examined. Five studies measuring outcomes for receptive language were identified. The results indicated that there was no conclusive evidence for the efficacy of oral language comprehension interventions (standardised mean difference = −0.04.).

In a systematic review by Cirrin and Gillam (2008) that focused on spoken language disorders in school-aged children (4–14 years), six studies were identified measuring receptive language outcomes. Four of these interventions had positive effects on oral language comprehension. Effect sizes were reported in two of the four effective interventions and they ranged from d = 1.1 to 1.3, indicating a large effect.

The only review focusing solely on oral language comprehension examined interventions in 2–16-year-old children with mixed receptive-expressive language impairment (Boyle et al., 2010). Ten studies were identified which were not included in previous reviews. Six out of the ten studies indicated efficacy. Effect sizes were not reported, except for one study which had a standardised effect size of 1.07, indicating a large effect size.

To conclude, the evidence regarding the efficacy of therapy techniques targeting oral language comprehension is mixed. Reported effect sizes have varied between no effect and large effects. In addition, in many cases the effect sizes have not been reported, even though this information is crucial for understanding the expected effects of an intervention when targeting oral language comprehension. To maximise outcomes, it is important to understand the efficacy of different therapy techniques, and the size of their effects.

### Level of evidence in oral language comprehension intervention studies

Intervention studies can be categorised by the level of evidence to evaluate the quality of the evidence. By quality of evidence we refer to “the methods used by the investigators during the study to minimise bias and control confounding within a study type (ie how well the investigators conducted the study)” (National Health and Medical Research Council (Australia), 2000, p. 14). The level of evidence informs researchers and practitioners regarding how much confidence they can have in the results. One such categorisation is the classification by the National Health and Medical Research Council (NHMRC) (National Health and Medical Research Council (Australia), 2000). It is a six-grade classification where systematic reviews of randomised controlled trials (RCTs) represent the highest level of evidence, and intervention studies with a pre-test/post-test design without experimental control present the lowest level of evidence.

Previous reviews have included a range of study designs (Boyle et al., 2010; Cirrin & Gillam, 2008; Law et al., 2003, 2004). How the level of evidence was evaluated varied between the studies. The systematic review and meta-analysis of RCTs conducted by Law et al. (2003, 2004) is considered to present the highest level of evidence. The systematic review by Cirrin and Gillam (2008) included intervention studies with the following study designs: RCTs, meta-analyses and systematic reviews of RCTs, nonrandomised comparison studies, and multiple-baseline single-subject design studies. The level of evidence of the included studies was evaluated by critical appraisal points and the authors state a moderate degree of confidence in the results with few exceptions. Boyle et al. (2010) classified articles either as RCTs or phase I and small-scale trials, but the level of evidence was not evaluated further. This variation in reporting the level of evidence creates uncertainty in the confidence researchers and practitioners can have in the results of oral language comprehension interventions. To enable judgements about the quality of the evidence and improve confidence in the results, it is important that the level of evidence is presented clearly in different studies and evaluated in reviews.

### Rationale and aim of this review

Despite the obvious need for oral language comprehension interventions, they have received little attention, and research in the area is scarce (Boyle et al., 2010). The Royal College of Speech and Language Therapists has listed the top-ten research priorities for DLD (Royal College of Speech and Language Therapists, 2019). In this list, the fourth research priority is “Effective interventions targeting receptive language for individuals with DLD”. These priorities were decided as a collaborative work between speech and language therapists, service-users, and parents, indicating that there is a real clinical need for more information on targeting oral language comprehension. In addition, the reviews of Law et al. (2003, 2004), Cirrin and Gillam (2008), and Boyle et al. (2010) all concluded there is a need for more research on comprehension interventions. The number of interventions targeting oral language comprehension included in each review was ten or fewer. There is also a need for an updated review including more recent research. In addition, no reviews have focused solely on oral language comprehension interventions in children with language disorders and difficulties aged 8 years and younger, even though children of this age with oral language comprehension difficulties form a common client group in speech and language therapy. In a Europe-wide survey, answered by more than 5000 speech and language therapists and other professionals managing children with DLD, 75% of the children who received interventions were up to 81 months (6.75 years) old (McKean et al., 2019). Thus, the age group eight years and younger was chosen to be the target population of the present review so that the study would capture the most common age group receiving speech and language therapy services, and interventions intended for them. Further, for an exemplar of their clients, 67% of the professionals answering the survey chose a child with difficulties in both receptive and expressive language (McKean et al., 2019). This indicates that children with difficulties in oral language comprehension form a large group within those receiving services. In addition, there are no reviews combining knowledge on intervention focus, efficacy, and level of evidence of oral language comprehension intervention studies. This information would improve understanding of the areas to be targeted when improving oral language comprehension, the efficacy of targeting a specific area of oral language comprehension, and how much confidence clinicians and researchers can have in the results. In short, the present review adds to the information needed to provide the best interventions possible for children with difficulties in oral language comprehension. To conclude, the aim of this study was to identify interventions targeting oral language comprehension in children aged 8 years and younger with language disorders or difficulties, and to examine the intervention focus, efficacy, and level of evidence in these intervention studies.

## Methods

### Study design of the present review

Prior to conducting this study, a preliminary search of the literature was carried out to explore the current research on oral language comprehension interventions. Relatively few RCTs were identified. Because of this, it was decided to include both RCTs as well as studies conducted with other research designs, such as pseudorandomised, time series, and pre-test/post-test designs in the present review. A systematic scoping review was chosen as the study design as the aim was to provide a descriptive article on the matter. Further, the limited amount of research on the topic indicated that in order to gain an overview of the matter, a relatively wide age group was warranted. In a field that has not been examined previously, or where little research has been done, scoping review designs are justified (Arksey & O’Malley, 2005). Scoping reviews differ from systematic reviews in several ways. They often have a broader research question than systematic reviews, inclusion/exclusion can be developed post hoc, quality of studies (i.e. quality of evidence) is not an initial priority, they may or may not include data extraction, synthesis is more qualitative than quantitative, and they are used to identify parameters and gaps in the research literature (Armstrong et al., 2011). Scoping reviews thus provide a broad map of the existing literature or evidence base of the desired field, as they can include studies with varying levels of evidence (Arksey & O’Malley, 2005; Armstrong et al., 2011). In particular the possibility of a qualitative synthesis on the broad topic was considered to meet the needs of the present study.

A ﬁve-stage methodological approach for scoping reviews has been incorporated into the Cochrane Public Health Review Body Guidance (Armstrong et al., 2011). This approach consists of: (1) identifying the research question; (2) identifying relevant studies; (3) study selection; (4) charting the data; and (5) collating, summarising, and reporting the results. In the field of speech and language therapy, the systematic scoping review protocol has been successfully used to research, for example, speech and language therapists’ public health practice (Smith et al., 2017). Although scoping reviews do not usually assess the quality of studies (Armstrong et al., 2011), in the present review the studies were classified by the level of evidence to enable researchers and practitioners to make judgements about the robustness of the study design and the confidence one can have in the results.

### Identifying the research question

The research questions were identified using the PICO framework (Schardt et al., 2007). The target population in this review were children with language disorders or difficulties. As the origin of the difficulties in oral language comprehension vary, the diagnoses of the included participants were not limited only to DLD. Other diagnoses, such as developmental delay, were also included as long as the child had language difficulties, in order to obtain an overview of the oral language comprehension interventions used. The target interventions were those aiming to improve oral language comprehension. No comparison treatment was chosen. Outcomes were children’s oral language comprehension skills. The research questions were:

1. Which interventions target oral language comprehension on its own or with expressive language in children 8 years and younger with language disorders or difficulties?
2. What is the focus of these interventions?
3. What is the efficacy of these interventions?
4. What is the level of evidence of these intervention studies?

### 2.3 Identification of relevant studies

Studies were identified from the following sources: Web of Science, Scopus, ERIC, LLBA, EBSCOhost, PsycINFO, Ovid, and PubMed. The search words were:

Intervention OR rehabilitation OR therapy OR treatment OR training OR enhanc* OR improv*

AND comprehen* OR receptive

AND language impairment* OR language disorder* OR language difficult*

AND child* OR adolesc* OR preschool OR school^2^

NOT aphasi* OR autism.

The detailed information on literature search is available upon request. Further studies were identiﬁed through reference lists of reviews identified during the database searches and included articles. Inclusion criteria are presented in Table 1.

**Table 1.** Inclusion criteria of the studies.

Participant ages were ≤8 years
Participants had a language disorder or language difficulties
Participant's language difficulties manifested in oral language comprehension or in receptive and expressive language
Participants had no sensory impairments
Study examined the effects of an intervention method targeting oral language comprehension independently or along with expressive language
Study had a detailed description of the intervention method used
Study had at least one assessment measure executed both before and after the intervention
Study was published in a peer reviewed journal
Study was published between the years 1996–2019
Study was published in English
Study was an intervention study reporting original results or a systematic review with or wihout a meta-analysis

### Study selection

**Figure 1.**
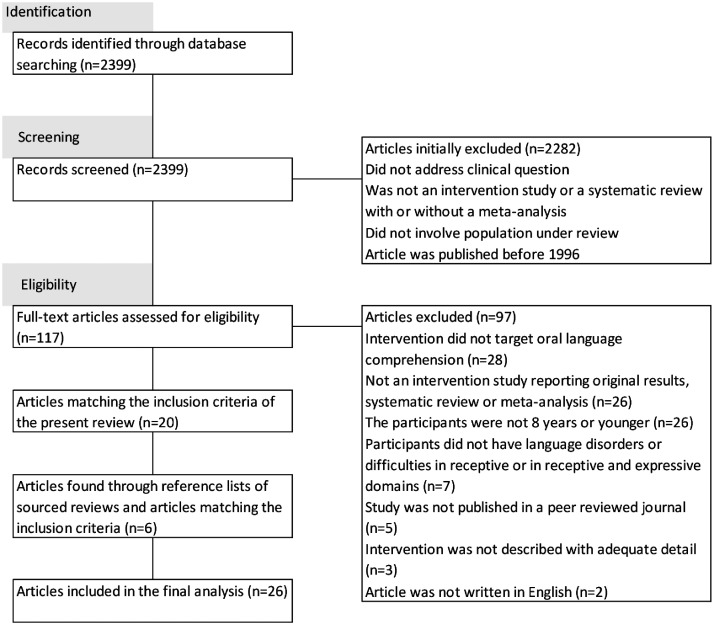
CONSORT flowchart: Identification of articles in this review.

The initial search was conducted in November 2016 and yielded 2265 results. Based on the screening of titles and abstracts, 102 articles were chosen for further inspection, and the full-text versions were obtained. Based on the full text, 15 articles were considered to meet the inclusion criteria. From the reference lists of sourced articles, an additional four articles were identified as eligible. An update search was conducted in January 2019 with the same search parameters as in the initial search. Of the 134 new results, 15 were chosen for further inspection based on the title and abstract. Based on the full text, five articles were considered to meet the inclusion criteria. From the reference lists of included articles, another two articles were included. The total number of articles matching the inclusion criteria in this review was thus 26. These 26 articles contained 25 studies. The inclusion and exclusion process of articles is summarised in Figure 1, adapted from CONSORT guidelines (Schulz et al., 2010). For simplicity, the initial and update searches are treated as one in the CONSORT flowchart.

### Charting the data

The data were charted using Excel software. The following data were extracted: author(s), year of publication, title, number of participants in experimental and control groups, participants’ age, diagnoses, therapy techniques, total intervention hours, intervention duration, provider of the intervention, main results, maintenance phase, generalisation, mention of bias, focus of intervention, effect size, and level of evidence. Total intervention hours were not always stated in the studies; in these cases, they were calculated for the purposes of this review based on the available information in the original studies or by contacting the authors. The provider of the intervention was classified based on who delivered the intervention to the child. When the providers were parents, they were first trained by a professional, such as speech and language therapist (SLT). The description of the intervention was considered sufficiently detailed if it could be categorised by the intervention focus.

### Collating, summarising, and reporting the results

The studies targeted oral language comprehension independently or along with expressive language. In the present review, only the results for oral language comprehension were taken into consideration and reported. It should be noted that many of the included studies reported positive effects on expressive language. Further, in the present review, the results are examined as a whole, but also separately for preschool and school-age children. Here, preschool-age is considered to be from 1 to 4 years and school-age from 5 to 8 years. The age groups were examined separately to see whether the focus of intervention, efficacy, or level of evidence differ between these two age groups. There were 9 studies on preschool children, 6 on school-age children, and 10 including both preschool and school-age children (see Tables 3 [Table table4-2396941520946999]to 5).

#### Focus of intervention

The studies were categorised by the area targeted in the intervention, i.e. the intervention focus. The classification of the intervention focus was generated based on the information in the studies identified in the search. If the target of the intervention was not explicitly stated, the best fitting intervention focus was chosen based on the information presented in the study. The employed therapy technique did not define the intervention focus. The categorisation was done based on where the change was expected to happen, i.e. in the skills or processes of the child or in the child’s surroundings. Three intervention foci were identified: modifying the communicative environment of the child, targeting the child’s language, and targeting the child’s language processing. The intervention foci is presented here from youngest to oldest.

Interventions were categorised as aiming to *modify the communicative environment of the child* when the target was to make the communicative environment more supportive for language development. The interventions were grouped as modifying the communicative environment when change was expected to be seen in the behaviour of the people communicating with the child. The assumption was that the new behaviour or communication skills learned during the intervention were also used after the intervention. This, in turn, was intended to improve the child’s language skills. All parent-implemented interventions were grouped into this category. Professional-implemented interventions were also included in this category if the language used by the professionals was one of the intervention targets, and if these professionals, such as day-care personnel or teachers, were also in contact with the child after the speech and language therapy intervention ended.

Interventions were categorised as *targeting the child’s language* when the focus was one or more of the language components contributing to oral language comprehension, i.e. when they aimed to improve an aspect of the child’s receptive language. Targeted areas in the included studies were receptive vocabulary, receptive morphosyntax, narrative comprehension, and inferential language. Interventions were carried out by someone other than the primary carer or another person (permanently) in the child’s surrounding: a professional, either a speech and language therapist or another professional under their guidance. Sometimes the parents were partly involved or received homework, like in the study of Wake et al. (2013). The focus of these interventions was considered to be only in the child’s language skills.

Interventions were categorised as *targeting the child’s language processing* when they aimed at improving more general language processing skills, such as processing auditory verbal signals, rather than directly targeting language skills. The improved language processing skills were then hoped to improve language skills. Interventions which were interpreted to aim at compensating poor language processing skills, such as narrow verbal working memory, were also categorised into this group. This is the case with mental imagery. In mental imagery, children are taught to think in pictures which will help understanding and remembering. The aim is not directly any of the language components contributing to oral language comprehension, but to teach children a metacognitive strategy which will help in coping with current language processing skills. It was interpreted that the main aim of mental imagery was to reduce the burden of verbal working memory by transferring auditive information into visual form. Hence it was considered to belong to the language processing group. Interventions targeting language processing were carried out by clinicians. Computerised training was also used in this intervention focus.

#### Efficacy

Intervention efficacy was classified by the information presented in the included articles. The magnitude of efficacy is reported by effect size. The studies used Cohen’s d (d), Hedges’ g (g), or omega squared (ω^2^) to report the effect size. In Cohen’s d and Hedges’ g, very large effect size is 1.2 or higher, large 0.8, medium 0.5, and small 0.2 (Cohen, 1988; Sawilowsky, 2009). In omega squared, large effect size is 0.15 or higher, medium 0.06, and small 0.01. If effect size was not calculated, but researchers reported improvement in comprehension skills, the efficacy was recorded as “reported benefits.” Some interventions reported no effect on language comprehension skills of the participants. The six categories of efficacy in the present review are thus: (1) very large effect; (2) large effect; (3) medium effect; (4) small effect; (5) reported benefits; and (6) no effect.

#### Level of evidence

The studies were sorted into six categories based on the quality of the evidence using the classification of the National Health and Medical Research Council, NHMRC (National Health and Medical Research Council (Australia), 2000). The NHMRC guidelines are developed by multidisciplinary committees that follow a rigorous evidence-based approach. The NHMRC classification was chosen because it is well-known and reliable, with distinct categories. The designation of levels of evidence is presented in Table 2.

**Table 2.** Designation of levels of evidence according to the National Health and Medical Research Council.

Level of evidence	Study design
I	Evidence obtained from a systematic review of all relevant randomised controlled trials.
II	Evidence obtained from at least one properly-designed randomised controlled trial.
III-1	Evidence obtained from well-designed pseudorandomised controlled trials (alternate allocation or some other method).
III-2	Evidence obtained from comparative studies (including systematic reviews of such studies) with concurrent controls and allocation not randomised, cohort studies, case-control studies, or interrupted time series with a control group.
III-3	Evidence obtained from comparative studies with historical control, two or more single arm studies, or interrupted time series without a parallel control group.
IV	Evidence obtained from case series, either post-test or pretest/post-test.

**Table 3.** Intervention studies: Modifying the communicative environment of the child.

Study	Focus of intervention	Level of evidence	N	Control group	Age, years	Diagnosis	Therapy technique and provider	Total hours	Outcome measures	Results	Efficacy	Maintenance	Generalisation
Roberts & Kaiser, 2011	Parents' communication strategies*	Systematic review, III-2	680		1–6	Primary & secondary language imparments	Several techniques increasing parent–child turn taking & improving parent responsiveness to communication; parents	Professional guided parents for mean 23 h, parental use not reported	Several different	Oral language comprehension improved	Small (g=.35 in receptive language)	n/a	n/a
Roberts & Kaiser, 2015	Parents' communication strategies*	III-1	45	52 usual care	2–3	Language delay	Enhanced milieu teaching, teach-model-coach-review; parents	Professional guided parents for 28 h + parents with children 204	PLS-4: Auditory Comprehension subscale	Oral language comprehension improved	Small (d=.27 PLS-4: Auditory Comprehension subscale)	n/a	Experimental group children were less likely to meet the criterion for language delay
Roberts & Kaiser, 2012	Parents' communication strategies*	III-1	16	18 no int. + 28 td	2–3	Language Impairment	Enhanced milieu teaching, teach-model-coach-review; parents	Professional guided parents for 24 h, parental use not reported	PLS–4: Auditory Comprehension subscale	Oral language comprehension improved	Small (d=.46 PLS-4: Auditory Comprehension subscale)	n/a	n/a
Van Balkom et al., 2010	Parents' communication strategies*	III-1	11	11 DCI	2–3	Language delay	PVHT; attachment, referencing, relevance and connectivity of language; parents	Professional guided parents for 9 h, parental use not reported	RLDS	No significant difference between the two interventions	Effect size n/a, reported benefits	3 months & again in 2 years	Indicative signs that less support was needed in school after two years for PVHT group
Colmar, 2011	Parents' communication strategies*	III-1	7	7 no int.	3–5	Language delay or difficulties	Pausing & expanding in shared book-reading & everyday situations; parents	Professional guided parents for 1 h, Parental use not reported	TELD3	No significant results to oral language comprehension	Results were not significant, large (d=.86 in TELD3 RLQ)	n/a	Parents reported positive changes in children's skills
Colmar, 2014	Parents' communication strategies*	III-2	11	12 no int. + 13 td	4–5	Language delay or difficulties	Pausing & expanding in shared book-reading & everyday situations; parents	Professional guided parents for 1 h, parents with children 9–28	TELD3; PPVT3	Oral language comprehension improved	Large to very large (d=1.67 for receptive language in TELD3, d=.80 in PPVT3)	n/a	Parents reported positive interactions & progress in everyday situations
Breit-Smith et al., 2017	Clinician's use of language facilitation strategies; Receptive vocabulary	IV	6	0	3–4	Language impairment	Interactive book reading with expository books & language facilitation strategies; practitioners (SLTs and teachers)	9	Researcher designed receptive vocabulary assessment	Significant increases in understanding of expository text & language skills	Effect size n/a, reported benefits	n/a	n/a
Hargrave et al., 2000	Teachers use of elements associated with dialogic reading; Receptive vocabulary*	III-2	36 participants in total, division into experimental and control group not known. Control group: typical practice		3–5	Children with poor vocabulary skills	Dialogic reading; teachers	3 at day care, 3 at home (78% of families participated in home activities)	PPVT-R	No effects on oral language comprehension	No effect	n/a	n/a

Note: *= The intervention focused both on oral language comprehension and expressive language; g = Hedges' g; n/a = Information not available; PLS-4 = Preschool Language Scale; d = Cohen's d; no int. = No intervention; td = Typically developing children; DCI = Direct Child Language Intervention; PVHT = Parent-based Video Home Training; RLDS = Reynell Language Development Scales; TELD3 = Test of Early Language Development; RLQ = Receptive language quotient; PPVT3 = Peabody Picture Vocabulary Test, 3rd edition; PPVT-R = Peabody Picture Vocabulary Test–Revised.

A properly designed randomised controlled trial was defined as a RCT with random allocation, blinded assessors after the intervention and reported attrition. A well-designed pseudorandomised trial was defined as a trial with blinded assessors after the intervention and reported attrition. If the study lacked the required elements, it was designated to a category that was one level lower. All studies using time series design without a control group were designated to level III-3. To be classified as time series design, at least three measurement points were required before the intervention. Studies without a control group, with two or more intervention groups that were not compared with each other, were considered to be single arm studies, and were designated to level III-3. A study was categorised as pre-test/post-test design when there was only one intervention group and no control group. Studies with only post-test measures were not included in the present review (see Table 1).

Studies designated level I were considered to have a very high level of confidence in the results. Results of studies at level II were considered to have a high level of confidence, whereas studies from levels III-1 to III-3 were considered to have a moderate level of confidence. Level IV studies were considered to provide indicative level of confidence considering their results.

#### Reliability

In order to eliminate researcher bias and to examine the reliability of categorization a reliability check on focus of intervention and level of evidence was conducted. The reliability check was conducted by a PhD candidate in speech and language pathology who was not associated with the present review. A randomly chosen 20% of the studies (5) were categorised. The categorisation of focus of intervention matched each other in four out of five studies and with discussion a consensus was reached. For level of evidence, the first-round reliability check yielded a match only for two out of five studies. The definitions used in the present study were refined concerning, for example, what was considered to be a properly designed randomised controlled trial or well-designed pseudorandomised trial, and the current criteria were created. After this, another 20% of the studies were randomly chosen for reliability check. The first author also re-evaluated all of the studies included in the present review in order to match the refined criteria. A second-round reliability check yielded a match of 100 percent.

## Results

### Description of the studies

The included studies tested the efficacy of a specific intervention method (see Tables 3 [Table table4-2396941520946999]to 5), compared two or more intervention methods, tested different forms of therapy delivery (specialist intensive, nursery-based, and no intervention) (Gallagher & Chiat, 2009), or gathered information from previous studies into a systematic review (Roberts & Kaiser, 2011). Wake et al. (2015) reported the follow-up results of a previous study (Wake et al., 2013). In the present review, these two articles are treated as one study. Of the 25 studies, 22 targeted both oral language comprehension and expressive language. Two studies focused only on oral language comprehension, and one study on both oral language comprehension and reading comprehension. Altogether 2460 participants were included in the 25 studies. The children’s ages varied between 1;3 and 8;5 (years; months). In 36% (9/25) of the studies, the participants were diagnosed as having SLI, LI, or DLD. In 48% (12/25) of the studies, participants were described as having a language delay, language difficulties, language delay/difficulties, high risk for reading comprehension difficulties, low listening comprehension, low receptive vocabulary skills, or poor expressive and receptive language skills. In 16% (4/25) of the studies, there was a large variation in the diagnoses of the participants. In these studies, some of the children had specific or primary language impairment, and some had nonspecific or secondary language impairment. In addition, in one of the studies subjects had the following diagnoses: SLI, autism spectrum disorders, developmental delay, and Down syndrome.

### Focus of intervention

#### Modifying the communicative environment of the child

The studies aiming to modify the communicative environment of the child (8/25, 32%) targeted parents’ communication strategies or clinicians’ language (Table 3). One systematic review and seven intervention studies were identified. All but two studies targeted parents’ communication strategies. The systematic review focused on 1–6-year-old children with primary or secondary language impairment (Roberts & Kaiser, 2011). Several different methods were used in the included studies, all of which focused on increasing parent–child turn taking or improving parents’ sensitivity as communication partners. A positive effect with small effect size on oral language comprehension was observed.

The individual intervention studies targeting parents’ communication strategies were conducted with 2–5-year-old children and their parents. The therapy techniques used were enhanced milieu teaching (Roberts & Kaiser, 2012, 2015), parent-based video home training (van Balkom et al., 2010), as well as pausing and expanding in shared book reading and in everyday situations (Colmar, 2011; Colmar, 2014). Enhanced milieu teaching (EMT) is a conversation-based therapy technique where the child’s interests are used as opportunities to model and prompt language use in everyday contexts. In EMT, the caregiver targets were, among others, matched turns, responsiveness, and expansions. In parent-based video home training, parents were trained in attachment, referencing, relevance, and connectivity of language. In pausing and expanding in shared book reading, and in everyday situations, the parents were advised to pause to allow the child to choose or initiate a topic of interest to them, and to ask an open question related to the child’s chosen topic. In one of the two studies targeting clinician’s communication strategies, speech and language therapists and teachers worked together with 3–4-year-old children using interactive book reading with expository books and language facilitation strategies (Breit-Smith et al., 2017). The language facilitation strategies included asking questions that focused children’s attention on the expository structure and asking children to make inferences. The children were also provided support through extending utterances and helping children construct responses to questions. Dialogic reading was used in the other study targeting clinicians’ language (Hargrave & Sénéchal, 2000). The children were encouraged to participate in reading, adults provided feedback to the child, and adapted their reading style to the child’s growing linguistic abilities. All but one (Hargrave & Sénéchal, 2000) of the interventions targeting the communicative environment of the child indicated positive effects on children’s oral language comprehension. The intervention of Hargrave & Sénéchal (2000), which reported no effect on oral language comprehension, had one of the lowest numbers of total hours (6) of the interventions with this focus. Maintenance was reported only in one study (van Balkom et al., 2010). Two years after the intervention, children who had received parent-based video home training were less likely to have DLD or to be placed in a special school for speech–language disordered children than the comparison group children receiving direct child language intervention. Similarly, in the study by Roberts & Kaiser (2015), the children whose parents were advised in their communication strategies were less likely to meet the criterion for language delay after the intervention.

#### Targeting the child’s language

Targeting some aspect(s) of the child’s language was the most common focus (15/25, 60%). Eleven studies targeted one or two aspects of the child’s language (receptive vocabulary, receptive morphosyntax, narrative comprehension, or inferential language), and four language programs aimed to improve several different language skills together (Table 4).

**Table 4. table4-2396941520946999:** Intervention studies: Targeting the child's language.

Study	Focus of intervention	Level of evidence	N	Control group	Age, years	Diagnosis	Therapy technique and provider	Total hours	Outcome measures	Results	Efficacy	Maintenance	Generalisation
Pollard-Durodola et al., 2011	Receptive vocabulary*	III-2	81	67 typical practice	4–5	Children with low receptive vocabulary skills	Words of Oral Reading and Language Development (WORLD) intervention; preschool teachers	20	Researher designed receptive picture vocabulary test of taught words; PPVT-III	Both groups improved in general vocabulary, intervention group demonstrated greater growth in taught words	Effect size n/a, reported benefits	n/a	n/a
Davis et al., 2016	Receptive vocabulary*	III-3	23	0	3–7	10 ASD; 3 DD; 5 DS; 5 SLI	Mixed storybook and play vocabulary intervention; clinician (SLT)	Varied according to how fast the children learned ASD 15; DD 10; DS 20; SLI 12	Reseacher designed probes measuring receptive vocabulary	Children's receptive vocabulary grew	Effect size n/a, reported benefits	n/a	n/a
Zens, Gillon & Moran, 2009	Receptive vocabulary*	III-1	9 + 10	19 td	6–8	SLI	Phonological awareness and semantic intervention or the same intervention in reversed order; SLT-researcher	24	Word-learning probe	No effects on oral language comprehension	No effect	n/a	n/a
Gallagher & Chiat, 2009	Receptive vocabulary & morphosyntax*	II	8	8 nursery based model of intervention + 8 no int.	3–4	SLI	Modelling, sentence recasting, elicited imitation; SLT	90–96	RDS III comprehension subtest; BPVS	Comprehension of morpho-syntax and vocabulary improved	Very large (d=1.72 in RDS III, d=2.24 in BPVS)	n/a	Changes in attention, listening and play skills.
Phillips, 2014	Receptive vocabulary & morphosyntax*	III-3	197 in 12 groups	0	3–8	High risk for reading difficulties	Providing situations on targeted structures; para-professionals	16	Researcher designed listening-comprehension assessment	Listening comprehension improved	From small to large (on average between .29 and .54 d for each grade)	n/a	n/a
Camarata et al., 2009	Expressive language targeted, but oral language comprehension of interest; Morphosyntax	III-1	21	6 no int.	2–3	SLI	Imitation, modelling, conversational recasting and milieu teaching; clinician	24	PLS-3: Auditory Comprehension Quotient	Oral language comprehension improved	Medium (d=.78 in PLS-3)	n/a	Expressive skills enhancement generalised into receptive skills
Calder et al., 2018	Morphosyntax*	III-3	3	0	6–7	DLD	Shape Coding and implicit approaches; SLT-researcher	8	TROG-2	Statistically significant & positive results in 2 participants, clinically but not statistically significant results in 1	Effect size n/a, reported benefits	5 weeks, receptive morpho-syntax was not evaluated	n/a
Riches, 2013	Morphosyntax*	III-3	2	0	8	SLI	Providing situations on different passives, constructional grounding & construction conspiracy; SLT & SLT-student	2,5	Researcher designed listening-comprehension assessment	Oral language comprehension improved	Medium to very large (child 1 d=1.8, child 2 d=.71)	6 weeks, results remained	Relatively poor
Popescu et al., 2009	Narrative comprehension*	IV	3	5 no int. + 7 td	7	SLI & nonspecific LI	NBLI: listening, retelling with scaffolding, imitating & co-generating stories; clinician	12	Test of Narrative Language: narrative comprehension. Results not compared to control group.	Narrative comprehension seems to have improved	Effect size n/a, reported benefits	n/a	n/a
van Kleeck et al., 2006	Oral language comprehension; Infrential and literal language	III-2	15	15 no int.	3–5	LI	Shared reading; graduate and undergraduate research assistants	4	PPVT–III	Literal language comprehension improved	Large (ω²=.16 in PPVT–III)	n/a	n/a
Desmarais et al., 2013	Oral language comprehension; Inferential language	IV	16	0	4–6	SLI	Dialogic reading; child's own SLT	8 (from which 3 dial. reading)	Researcher designed listening-comprehension assessment	No significant effects on oral language comprehension	Effect size n/a, reported benefits	6 weeks	n/a
Bowyer-Crane et al., 2008	Language programme: different linguistic skills*	II	72 oral language	69 reading with phonology	4	Language difficulties	Direct instruction to develop vocabulary, inferencing, expressive language and listening; teaching assistant	42	Recordings of stories taken from NARA II:	No difference between the two groups on oral language comprehension	No effect	5 months	n/a
Fricke et al., 2017	Language programme: different linguistic skills*	II	132 (30 weeks intervention), 133 (20 weeks intervention)	129 waiting controls	3–5	Language difficulties	Nuffield Early Language Intervention; teaching assistant	30 week group max. 48h, 20 week group max. 38h	Modification of YARC to assess narrative comprehension; CELF: Sentence Structure subtest; BPVS	30 weeks: Narrative comprehension improved, no effects on receptive vocabulary or receptive grammar; 20 weeks: No effects	30 week group: Small (d=.39–.46 in modification of YARC to assess narrative comprehension)	6 months, results remained	n/a
Hagen et al., 2017	Language programme: different linguistic skills*	III-2	157	144 usual care	4	Language difficulties	Dialogic reading & instruction in vocabulary, narratives and grammar; preschool teachers	21	TROG-2; BPVS-II; researcher designed test of taught vocabulary & narrative comprehension tests	Understanding of grammar, receptive vocabulary & short narrative comprehension improved	Small to medium (d=.58–.64 in TROG-2; d=.45–.56 in BPVS-II; d=.29–.47 in taught vocabulary; d=.57–.75 in narrative comprehension)	7 months, results remained	Generalisation of skills was seen in improved results in standardised tests
Wake et al., 2013 & 2015	Language programme: different linguistic skills*	II	92	100 usual care	4–5	Language delay	Focusing on vocabulary, grammar, narratives, phonological awareness/preliteracy skills; language assistants & parents	18	CELF-P2: Sentence Structure, Concepts and Following Directions, Word Classes	No difference between the two groups on oral language comprehension	No effect	1 year & 2 years	Parents reported changes in their own and theirs child's communication skills

Note:*= The intervention focused both on oral language comprehension & expressive language; PPVT-III = Peabody Picture Vocabulary Test-III; n/a = Information not available; ASD = Autism spectrum disorder; DD = Developmental disability; DS = Down Syndrome; td = Typically developing children; no int. = No intervention; RDS III = Reynell Developmental Scales; BPVS = British Picture Vocabulary Scales; d = Cohen's d; PLS-3 = Preschool Language Scale; TROG-2 = Test of Reception of Grammar 2; NBLI = Narrative-based language intervention; ω² = omega squared; NARA II = Neale Analysis of Reading Ability II; YARC= York Assessment of Reading for Comprehension; CELF-P2 = Clinical Evaluation of Language Fundamentals – Preschool.

**Table 5.** Intervention studies: Targeting the child's language processing.

Study	Focus of intervention	Level of evidence	N	Control group	Age, years	Diagnosis	Therapy technique and provider	Total hours	Outcome measures	Results	Efficacy	Maintenance	Generalisation
Center et al., 1999	Oral language comprehension; reducing the burden on verbal working memory	III-2	33	33	7–8	Low listening comprehension	Experimental group: mental imagery, both groups: listening comprehension instruction; researcher	4	NARA Listening & Reading Comprehension (read aloud); Byrne Listening Comprehension	Oral language comprehension improved	From small to large (NARA Listening d=.44, NARA Reading d=.48, Byrne d=.80)	n/a	Results generalised to reading comprehension
Fey et al., 2010	Processing of auditory– verbal signals *	II	7 FFW /NBLI & 7 NBLI /FFW	9 wait/ NBLI	6–8	SLI & non-specific LI	Fast ForWord (FFW); clinicians aided in the computerised training	FFW 40, NBLI 12	NLAI & Narrative Comprehension Composite	FFW did not enhance children's response to a conventional language intervention	No effect	n/a	n/a

Note: NARA = the Neale Analysis of Reading Ability; d = Cohens' d; n/a = Information not available; *= The intervention focused both on oral language comprehension & expressive language; FFW = Fast ForWord; NBLI = Narrative-based language intervention; NLAI = Test of Narrative Language.

*Receptive vocabulary* was targeted in 3–8-year-old children in three interventions. Words of Oral Reading and Language Development (WORLD) is a technique where shared book reading was intensified with three principles: building vocabulary through thematically and conceptually related book reading, bridging vocabulary by integrating informal and narrative texts, and building vocabulary by using explicit instruction in shared book reading (Pollard-Durodola et al., 2011). In mixed storybook and play vocabulary intervention the child and the clinician first viewed a picture book together while the clinician told a story about the pictures (Davis et al., 2016). During the play context, the clinician and the child interacted with a set of toys that matched the storybook theme and included the target vocabulary items. In phonological awareness and semantic intervention, the following tasks were practised: phoneme segmentation, phoneme blending, phoneme manipulation, tracking sound changes, reading real and non-words, and identifying main features and attributes of familiar words (Zens et al., 2009). Of these three studies, WORLD (Pollard-Durodola et al., 2011) and mixed storybook and play vocabulary intervention (Davis et al., 2016) indicated positive effects on receptive vocabulary. None of these studies reported maintenance or generalisation.

Both *receptive vocabulary and receptive morphosyntax* were targeted in 3–8-year-old children using modelling, sentence recasting, and elicited imitation (Gallagher & Chiat, 2009). In modelling the SLT produced models of target utterances which were repeated several times using a variety of visual stimuli. In sentence recasting the SLT produced correct models of utterances that the children had initiated. In elicited imitation, the SLT modelled an utterance related to a visual stimulus and requested the child to repeat the utterance. Offering multiple situations on targeted structures to respond to and produce at sentence and narrative levels was also used (Phillips, 2014). In this study, story-based and prop-based activities were designed to solicit interest and provide an authentic, academically-relevant topic of discussion for the interventionist and children. The intervention provided a “flooding” of exposure to each unit’s target syntax features. The results of these two studies indicated positive effects on children’s comprehension of morphosyntax and vocabulary, as well as listening comprehension. Further, the use of modelling, sentence recasting, and elicited imitation resulted in positive changes in attention, listening, and play skills.

*Receptive morphosyntax* was targeted in 2–8-year-old children using Shape Coding, i.e. explicit teaching of grammatical rules with visual support, together with implicit approaches (Calder et al., 2018); constructional grounding and construction conspiracy, i.e. using short structures as the basis for acquiring long structures, and encouraging analogies between partially overlapping constructions (Riches, 2013); or imitation, modelling, conversational recasting, and milieu teaching (Camarata et al., 2009). Here, imitation requires the participant to repeat the sentences after the clinician model. In modelling, the child listens to clinician production. In conversational recasting, the clinician follows the child’s verbal and nonverbal lead and provides an immediate response to them. The responses repeat the central meaning of the child’s utterance and the target structure in a conversational context. Milieu teaching incorporated aspects of conversational recasting and imitation, with a focus on following the child’s lead and elicitation of target structures through prompting and imitation. All the techniques which aimed to improve receptive morphosyntax had a positive impact on oral language comprehension, with reported benefits or effect sizes varying from small to very large. In a study by Camarata et al. (2009), expressive skills were targeted to determine whether oral language comprehension skills would improve together with expressive skills. Enhancement in expressive language did generalise into improvement of receptive skills. Of the studies targeting receptive morphosyntax, only in the study of Riches (2013) were the long-term results assessed. The results remained unchanged after six weeks, but the generalisation of skills was relatively poor.

*Narrative comprehension* of 7-year-old children was targeted using narrative-based language intervention (NBLI) (Popescu et al., 2009). In NBLI, the sessions involved: (1) listening to the story and retelling it with the clinician scaffolding story content and target grammatical forms; (2) imitation of sentences containing target forms found in the story; and (3) cogeneration of a novel story. Although NBLI was reported to support narrative comprehension, the effect size was not calculated.

*Inferential language* was targeted in 3–6-year-old children using dialogic reading (Desmarais et al., 2013) and shared reading (van Kleeck et al., 2006). Though the names differ, the content of these two interventions were alike: the therapist pauses the reading to ask literal and inferential questions, and provides cues to scaffold the expected response. Neither study found significant effects on inferential language, but Desmarais et al. (2013) reported benefits and van Kleeck et al. (2006) found a large effect on the comprehension of literal language.

Four different *language programs* were identified that targeted oral language comprehension in 3–5-year-old children. First, the Nuffield Early Language Intervention aimed to improve children’s vocabulary, develop narrative skills, encourage active listening, and build conﬁdence in independent speaking (Fricke et al., 2017). This was done using multisensory techniques and multicontextual approach with games and other activities in a group setting. Second, dialogic reading with instruction in vocabulary, narratives, and grammar (Hagen et al., 2017) aimed to improve language comprehension and active listening skills. In dialogic reading, the teacher asked questions on the content of the story to help children to draw inferences about the course of the story, why certain things happened, and the meanings of novel words. Third, in the oral language program providing direct instruction to develop vocabulary, inferencing, expressive language, and listening skills were used (Bowyer-Crane et al., 2008). Fourth and finally, vocabulary, grammar, narrative skills, and phonological awareness/preliteracy skills were targeted together in one study (Wake et al., 2015, 2013). The used therapy techniques were: vocabulary expansion; identifying word features, sentence structures and grammatical markers; following instructions and asking clarifying questions; shared book reading; teaching story grammar elements; and practising left to right reading, awareness of rhyme, letter sound connections, phoneme identity, and phoneme matching. Of the four language programs, the interventions by Fricke et al. (2017) and Hagen et al. (2017) indicated positive effects on children’s oral language comprehension skills. In both of these studies, the results remained after six months.

#### Targeting the child’s language processing

Interventions targeting the child’s language processing (2/25, 8%) focused on improving the processing of auditory–verbal signals or compensating narrow auditory memory (Table 5). Fast ForWord (FFW) is a language program that includes seven computerised listening games. These games include acoustically modified nonspeech and speech stimuli which aim to ameliorate the proposed inability to properly process the rapidly changing acoustic features of the speech stream (Fey et al., 2010). FFW was used to improve 6–8-year-old children’s processing of auditory–verbal signals, but was not found to have an effect on oral language comprehension. Mental imagery was used to aid oral language comprehension in 7–8-year-old children (Center et al., 1999). In this method, the children were explicitly taught to create visual images elicited by adults via questions and verbal guidance. In this way, the auditory information was transformed into a visual form, which was considered to reduce the burden on verbal working memory. Effect sizes of mental imagery intervention ranged from small to large and results generalised to reading comprehension.

The intervention foci were associated with the age of the participating children. In studies intended for preschool children (n = 9) the interventions focused on modifying the communicative environment (44%, 4/9) or on the child’s language (56%, 5/9). None of the interventions in children aged 4 years or younger targeted language processing. In the mixed group, including interventions for both preschool and school-age children (n = 10), 40% (4/10) of the studies modified the communicative environment, and 60% (6/10) targeted the child’s language. None of the interventions in this group targeted language processing. In school-age children, the interventions (n = 6) targeted the child’s language (67%, 4/6) or their language processing (33%, 2/6), but none targeted the communicative environment of the child.

### Efficacy of oral language comprehension interventions

Nearly half (48%, 12/25) of the included studies reported effect sizes from small to very large, indicating positive effects on oral language comprehension (Tables 3 [Table table4-2396941520946999]to 5). Reported effect sizes varied between d=.27 (small) and d = 2.24 (very large). The interventions indicating the most efficacy according to the effect size were: modelling, sentence recasting, and elicited imitation (Gallagher & Chiat, 2009); pausing and expanding in shared book reading and in everyday situations (Colmar, 2014); shared reading (van Kleeck et al., 2006); constructional grounding and construction conspiracy (Riches, 2013); mental imagery (Center et al., 1999); imitation, modelling, conversational recasting, and milieu teaching (Camarata et al., 2009); providing situations on targeted structures (Phillips, 2014); and dialogic reading and instruction in vocabulary, narratives, and grammar (Hagen et al., 2017). In 32% (8/25) of the studies, researchers reported benefits, but effect size was either not calculated or the results failed to reach statistical significance. In these studies, researchers reported improvements in children’s oral language comprehension skills or parents’ positive remarks on children’s language skills. Altogether 80% of the studies indicated thus positive effects on participants’ oral language comprehension. In the remaining 20% (5/25) of the studies, authors reported no effects on oral language comprehension.

All but one of the studies *modifying the communicative environment of the child* (88%, 7/8) indicated efficacy, meaning that most of the interventions with this focus had a positive impact on the children’s oral language comprehension. Half of the studies (4/8) reported effect sizes between small and very large, indicating that participants in all of these intervention studies had improved significantly in their skills, but the degree of change varied greatly. The authors reported benefits in 38% (3/8) of the studies with this focus. One of the studies indicated no effect on oral language comprehension (Hargrave & Sénéchal, 2000). In *targeting the child’s language,* the majority of the studies (80%, 12/15) indicated efficacy, and there was a large variation in the effect sizes. Effect sizes ranged from small to very large in 47% (7/15) of the studies. No effect size was calculated in 33% (5/15) of the intervention studies, but the authors reported benefits. The authors reported no effect on oral language comprehension in 20% (3/15) of the interventions targeting the child’s language. The studies t*argeting the child’s language processing* had the greatest variation in results, but also the smallest number of studies (two). One of the two interventions reported effect sizes from small to large, indicating efficacy (Center et al., 1999), whilst the other, using Fast ForWord, indicated no effect on oral language comprehension (Fey et al., 2010).

The efficacy of the interventions for preschool- and school-age children differ slightly (see Tables 3 [Table table4-2396941520946999]to 5). Of the interventions intended for preschool children, 89% (8/9) indicated efficacy measured by effects size or reported benefits. The interventions covering both preschool and school-age children indicated efficacy in 80% (8/10) of the studies. The interventions for school-age children indicated efficacy in 67% (4/6) of the studies.

Efficacy of interventions was examined also in the three different diagnostic category groups: (1) DLD, SLI, and LI; (2) language delay or difficulties; and (3) diverse disorder typologies. In the studies in which participants had SLI, LI, or DLD, 89% (8/9) indicated efficacy either by effect size or reported benefits (see Tables 3 [Table table4-2396941520946999]to 5). In the group of language delay or difficulties, 75% (9/12) of the studies indicated efficacy. In studies on participants with diverse disorder typologies, 75% (3/4) indicated efficacy.

### Level of evidence

The level of evidence (see Table 2 for designation of level of evidence) of the included studies varied between II and IV (Tables 3 [Table table4-2396941520946999]to 5). No systematic reviews including only RCTs were identified, meaning no study reached level I, or very high level of confidence in the results. The only systematic review identified (Roberts & Kaiser, 2011) also included pseudorandomised studies, and the level of evidence was designated III-2. The median of level of evidence in the included studies was III-2, as was the mode.

Of the included studies, 20% (5/25) were properly-designed RCTs and were at level II. Based on the categorisation used in the present study, one can have a high degree of confidence in the results of these studies in which the following therapy techniques were used: (1) modelling, sentence recasting, elicited imitation (Gallagher & Chiat, 2009); (2) Nuffield Early Language Intervention (Fricke et al., 2017); (3) focusing on vocabulary, grammar, narrative skills, and phonological awareness/preliteracy skills (Wake et al., 2013, 2015); (4) Fast ForWord (Fey et al., 2010); and (5) direct instruction to develop vocabulary, inferencing, expressive language, and listening skills (Bowyer-Crane et al., 2008). The first two studies found a positive effect on oral language comprehension whereas the last three indicated no effect.

In 24% (6/25) of the included studies, the level of evidence was III-1 (see Tables 3 [Table table4-2396941520946999]to 5). These were controlled trials which did not qualify for level II or pseudorandomised controlled trials comparing experimental and control groups to each other. The proportion of studies at level III-2 was 28% (7/25). These were pseudorandomised studies that failed to reach level III-1 or comparative studies with non-randomised allocation, and the only systematic review. Most studies were thus at an evidence level of III-2 or III-1. 16% (4/25) of the studies were time-series or single arm studies and were designated level III-3. Based on the categorisation of the present review, the results of level III-1 to III-3 studies (68%; 17/25) provide moderate confidence in the results. One can thus have moderate confidence in the results of most of the therapy techniques included in this review.

Another 12% (3/25) of the studies were at level IV. These were studies using pre-test/post-test design without experimental control. The results considering the therapy techniques on this level should be considered indicative. These techniques are: interactive book reading with expository books and language facilitation strategies (Breit-Smith et al., 2017); dialogic reading (Desmarais et al., 2013); and narrative-based language intervention (Popescu et al., 2009).

The interventions for preschool children have a slightly higher level of evidence than those for school-age children (see Tables 3 [Table table4-2396941520946999]to 5). The median of level of evidence of interventions for preschool children is III-1, mixed group, containing both preschool and school-age children, III-2, and school-age children III-2 and III-3. The mode of level of evidence in interventions for preschool children is III-1; for mixed group III-2; and for school-age children III-3. All the median and mode values presented here indicate moderate confidence in the results.

## Discussion

The purpose of this systematic scoping review was to identify interventions aimed at improving oral language comprehension in children of 8 years-of-age or younger, with language disorders or difficulties. Further, the aim was to examine the possible intervention foci, efficacy, and level of evidence of these interventions. Altogether, 25 studies including 2460 children were included. The interventions focused on modifying the communicative environment of the child, some aspect(s) of the child’s language, or the child’s language processing. Efficacy of the interventions varied from very large effect size to no effect. Of the studies included in the present review, 80% indicated positive effects on participants’ oral language comprehension. Level of evidence in the included studies varied between II and IV, suggesting high to indicative confidence in the results of the studies. The majority of the studies were at levels III-1 to III-3, indicating moderate confidence in the results of most of the studies. Although the evidence is still limited, the results of this systematic scoping review suggest that there are effective interventions to ameliorate problems in oral language comprehension of 1–8-year-old children with language disorders or difficulties.

### Focus of intervention

Three intervention foci were identified in this review: modifying the communicative environment of the child, targeting the child’s language, and targeting the child’s language processing. The latter two of these foci have been reported in earlier reviews (Boyle et al., 2010; Cirrin & Gillam, 2008; Law et al., 2003, 2004). However, the results of the present review identified modifying the communicative environment of the child as an important and effective intervention focus. Also maintenance and generalization, when reported in the studies, was good in interventions targeting parents’ communication strategies, which further emphasizes the rationale for modifying the communicative environment. The only systematic review included in this review focused on parent-implemented interventions. A recent systematic review and meta-analysis further confirms the positive effect parent-implemented interventions have on oral language comprehension skills (Roberts, Curtis, Sone, & Hampton, 2019). Modification of a child’s communication environment by guiding parents can thus be argued to have the strongest evidence level of the three intervention foci. Further, when the child is aged 18–24 months, the quality of parent-child interaction has long lasting effects on a child’s oral language comprehension skills — and also on child’s IQ — which can still be seen after ten years (Gilkerson et al., 2018). This emphasises the rationale for modifying the communication strategies of the people in the child’s surroundings. It would also be worth determining how other methods of modifying the communicative environment, besides guiding people in the child’s surroundings, would improve comprehension. An example would be analysing the effects of the use of visual support (pictures, signs) in the daily environment on the language comprehension skills of young children with language disorders or difficulties.

In this, and previous reviews, targeting the child’s language has been the most common focus. Targeting the child’s language processing has also previously been suggested as an intervention target (Cirrin & Gillam, 2008). However, interventions aiming to compensate current, often limited, language processing skills have not been mentioned when targeting a child’s language processing. These compensatory strategies, such as mental imagery, are important as language comprehension difficulties are persistent, and children with these difficulties need ways to cope with their challenges (Boyle et al., 2010).

Almost all the included studies targeted both receptive and expressive language. This is expected as interventions in young children can improve both expressive and receptive modalities (see for example Camarata et al., 2009; Roberts & Kaiser, 2011; Roberts et al., 2019) and the learning seems to be more holistic. The language programs targeting several different language skills in 3–5-year-old children reflect this view of holistic learning. In addition, exposing young children to optimal language seems to be enough to enhance the child’s skills. Providing a more optimal language environment through improving the communication strategies of the people in the child’s surroundings enhances language comprehension skills of children aged 1 to 6.

Based on the present findings, the focus of intervention is related to the age of the child. Though in both preschool and school-age children the interventions focused on the child’s language, only in children up to 6 years the interventions focused on modifying the communicative environment. The only interventions targeting the child’s language processing were intended for children from 6 to 8 years. As children grow older, the increasing cognitive abilities and language skills offer more possibilities for learning and the use of metacognitive strategies becomes possible.

### Efficacy of oral language comprehension interventions

The results presented here align with the results of two previous reviews (Boyle et al., 2010; Cirrin & Gillam, 2008), in that not all interventions provide desired outcomes, but there are interventions indicating efficacy. Based on the findings of the present review, the right question regarding efficacy of oral language comprehension interventions does not seem to be whether oral language comprehension interventions provide desired effect or not, but rather which interventions indicate efficacy in improving one or more areas of oral language comprehension, and what is the magnitude of the effect.

Efficacy of an intervention depends on several factors, one being the theoretical underpinnings of the intervention. Interventions indicating efficacy in the present review are based on acknowledged theories, such as social interactionist and usage-based theories (see for example Saldaña & Murphy, 2019). Social-interactionist, or socio-pragmatic, and usage-based theories agree that language is not an innate system, but is acquired. Language structures emerge from an interaction between the child and their environment. Saldaña and Murphy (2019) state that “Although these theories do not originally relate directly to intervention, they do provide an overarching framework that influences its orientation.” (p. 58). Thus, the theoretical framework of language and language acquisition of the clinician influences the choice of used interventions. Not all theoretical considerations of language are supported by research evidence and the efficacy of interventions based on these assumptions can be questioned. Therefore, if an intervention has no effect, one reason may be that the intervention is inherently faulty, i.e. there is a failure of intervention theory of concept (Rychetnik et al., 2002). Current evidence suggests that an attempt to modify auditory processing in such a way that oral language skills are improved, may be inherently faulty. An example of an intervention based on this assumption is Fast ForWord (n.d.). Fast ForWord was used in one study in this review with no effects on oral language comprehension (Fey et al., 2010). This aligns with a systematic review on Fast ForWord intervention which concluded that it has no effect on children’s language skills (Strong et al., 2011).

Another potential reason for an intervention being unsuccessful may be flaws in delivery, i.e. a failure in implementation (Rychetnik et al., 2002). Factors related to implementation include the provider, fidelity, and dose of the intervention. The provider has not been found to have a large effect on intervention efficacy (Law et al., 2003), and relatively little discussion has ensued on the role of fidelity in efficacy. The role of dose, by contrast, has been discussed extensively (see for example Justice, Logan, Jiang, & Schmitt, 2017; Schmitt, Justice et al., 2017). Oral language comprehension difficulties are considered resistant to intervention (Boyle et al., 2010), and it has been stated that oral language comprehension interventions should be prolonged and intensive (Hagen et al., 2017). The dose of intervention might explain, for example, why the intervention of Hargrave and colleagues (2000) had no effect on oral language comprehension although all the other interventions which focused on modifying the communicative environment of the child did: their intervention consisted of only six hours whereas all but one of the other interventions had at least nine, and in most cases, significantly more intervention hours. The effect of implementation, especially dose, in relation to efficacy was not examined in this review, but it should be examined in the future.

The research design used may also impact the assessment of intervention efficacy. When two active interventions are compared with each other, it can be difficult to detect efficacy, as one intervention must result in a larger effect than the other to detect a difference. Also, in interventions where the control group receives “treatment as usual,” it is more difficult to detect an effect than when the control group receives no intervention. In the present review, three studies compared experimental treatment with another kind of treatment (Bowyer-Crane et al., 2008; Gallagher & Chiat, 2009; van Balkom et al., 2010). Of these three studies, only one (Gallagher & Chiat, 2009) reported effect sizes indicating improvement in oral language comprehension. In the study in question, however, the intervention hours were significantly higher for the experimental group than for the control groups. The other two studies (Bowyer-Crane et al., 2008; van Balkom et al., 2010), with more comparable settings, found no difference between the experimental and the control intervention. When active interventions with similar dosage are compared to each other there is thus a risk that the evaluation of efficacy becomes more difficult and making conclusions on the results challenging.

The results of this review are in contradiction to the findings of the review of Law et al. (2003, 2004) which concluded that there is little evidence that interventions are effective for children with receptive difﬁculties. Previously mentioned factors in implementation and research design could partly explain the differences in results between this review and the systematic review of Law and associates. One of the five studies measuring outcomes of oral language comprehension in the review of Law et al. (2003) had a weekly intervention time of ten minutes, indicating that the implementation time might have been too short to improve children’s skills. Another two of the five studies compared active interventions with each other, making it harder to indicate efficacy. Furthermore, the review of Law and colleagues was conducted in 2003 and contained a very small number of studies targeting receptive language. New studies have been published since, which in turn affect the conclusions that can be drawn on the efficacy of oral language comprehension interventions.

The reported effect sizes in the studies included in the present review indicated a significant change in oral language comprehension of the children compared to the suggested benchmark of effect sizes in the literature. The expected effect size in 3–9-year-old children with language impairment receiving speech and language therapy during one academic year varies between g = 0.51–0.70, indicating medium effect sizes (Schmitt, Logan et al., 2017). In the studies included in the present review reporting effect sizes (48%), the effect sizes varied from small to very large. The mean duration of these studies was 14 weeks, which is less than half of an academic year. The reported efficacy of the included studies reporting effect size compared to the benchmark by Schmitt, Logan et al. (2017) suggests that these interventions have resulted in a substantial change in children’s skills. That is, during less than half of the time in the benchmark of Schmitt, Logan et al. (2017), 71% of the interventions reporting effect size have resulted in effect sizes of the same magnitude (medium) and higher (large and very large). The number of studies reporting effect size is small and the diagnoses varied, however, and these results should be interpreted with caution. It is too early to say much about the expected effect sizes regarding different interventions and intervention foci on oral language comprehension because of limited research evidence. The results do suggest, however, that when the intervention targets the communication strategies of the parents with an intervention lasting several hours, a small effect size on children’s oral language comprehension is expected.

It should be noted that it might be more difficult to gain a large effect size using a clinical test compared to a researcher created measure on practiced items. Clinical tests often measure a variety of skills and thus require greater learning or generalization of acquired skills to achieve similar effect sizes as seen in researcher-created measures of practised items. Therefore, it is likely that a large effect size is detected less often when a clinical test is used as an outcome measure. In light of this, the efficacy of the following interventions, measured with clinical tests, seem very promising: modelling, sentence recasting, and elicited imitation (Gallagher & Chiat, 2009); pausing and expanding in shared book-reading and in everyday situations (Colmar, 2014); shared reading (van Kleeck et al., 2006); mental imagery (Center et al., 1999); and imitation, modelling, conversational recasting and milieu teaching (Camarata et al., 2009).

Based on the present findings, there seems to be a small difference in the efficacy favouring interventions in young children. The percentage of interventions indicating efficacy in preschool children was 89%, in mixed groups 80%, and in school-age children 67%. This variation may be due to the difference in the persistency of language difficulties; language problems in children aged five and older are likely to persist (Stothard et al., 1998). The majority of the interventions for children aged 5 to 8 years did, however, indicate efficacy, which suggests that though the difficulties in this age group are persistent, they can be ameliorated. There seems to also be a small difference in the efficacy of interventions between the different diagnostic groups. The percentage of interventions indicating efficacy in the group with SLI, LI, or DLD was 89%; in the group of language delay or difficulties 75%; and in the group with diverse typologies 75%. It would seem more reasonable if the efficacy in the group of language delay of difficulties was higher than in the group of SLI, LI, or DLD, as the terms “language delay and difficulties” are used at a younger age than SLI, LI, and DLD, and as the difficulties of young children are ameliorated more often than those of children aged five and older. The number of studies in each group are small and drawing conclusions between the groups must be approached with caution.

In general, the present results on efficacy suggest that although oral language comprehension difficulties are considered resistant to intervention, they can be ameliorated with carefully chosen methods with solid theoretical background and good implementation.

### Level of evidence

The median (III-2) and the mode (III-2) of the level of evidence of the included studies indicate moderate confidence in the results. The level of evidence of the interventions for preschool children is slightly higher than for mixed-group or school-age children based on the median (III-1, III-2, and III-2 & III-3 respectively) and the mode (III-1, III-2, and III-3). This indicates a slightly higher confidence in the results of interventions for preschool children than for school-age children, though modes and medians of all the age groups fit into the category that is seen to provide moderate confidence in the results aligning with the overall results. However, the number of studies in each group was small and there is a clear need for further intervention studies targeting oral language comprehension skills with a high level of evidence. More properly designed RCTs and systematic reviews of specific intervention techniques are needed. At this stage, while there is still relatively little research on the topic and the level of evidence for some therapy techniques is low, clinicians also need to consider other factors besides level of evidence in clinical decision making. The theoretical underpinnings of an intervention may be used as one factor to aid in clinical decision making in the lack of research evidence. The stronger confidence we have in the theoretical background of the therapy technique in question, the more we may consider using therapy techniques which currently have a low level of evidence. Mental imagery represents one example of a therapy technique with little evidence but a reasonable theoretical background. Its effect on oral language comprehension of children aged 8 or younger is yet to be examined thoroughly. The theoretical assumption of the technique, however, makes sense: “the use of imagery training may provide poor comprehenders with an alternative route for integration of passage material by using an additional but non-phonological strategy” (Center et al., 1999, p. 242). The development of theories of treatment for language disorders are still in their infancy (Saldaña & Murphy, 2019), and in need of further research.

The designation of levels of evidence can be disputed in relation to their applicability for evaluating speech and language therapy interventions. Time series design, also known as single case experimental design, may be more feasible than RCTs when conducting intervention research, especially effectiveness research in a clinical speech and language therapy setting. In the NHMRC classification, time series design is ranked the second lowest level just before case studies, which have no experimental control. However, in the classification by the Oxford Centre for Evidence-Based Medicine (OCEBM Levels of Evidence Working Group, 2011), systematic reviews of N = 1 studies, and thus also time series studies, are considered to be as strong as systematic reviews of RCTs when examining treatment benefits. Research using time series design is therefore a valid method to increase knowledge on the efficacy of oral language comprehension interventions. When the knowledge from time series designs is summarised into systematic reviews, it presents the highest level of evidence. Overall, despite the need for more research employing robust research designs, researchers and practitioners can have moderate confidence in the results of the studies included in this review: oral language comprehension difficulties can be ameliorated with appropriate interventions.

### Limitations

The following limitations should be considered when interpreting the clinical implications of this review. The inclusion criteria used in the present review may have affected the results. The level of evidence of the studies included in this review varied. By including studies with a varying level of evidence, it is possible to detect a larger number of promising interventions. However, confidence in the results decreases at the same time. Although the categorization of level of evidence was used to minimise bias and control confounding within a study type, we acknowledge that, in the present review, the relative categorization used does not thoroughly examine the risk of bias in the individual studies included. The variability of the language profile of the participants in the included studies should also be considered. The participants did not comprise a homogeneous diagnostic group, which limits generalisation. The age range of the participants was also relatively wide, yet the trajectories of younger and older children differ (Bishop et al., 2017). This difference should be kept in mind when interpreting the results. In addition, only articles published between 1996 and 2019 were considered eligible. This may have excluded some relevant studies. Publications from the past two decades were, however, deemed adequate to provide an overview of the most recent intervention methods. The language of the interventions was mostly English, which limits the generalisability of these results to other languages. This is especially the case in interventions focusing on receptive grammar, since grammar varies between languages.

The amount and quality of the studies included in this review may also affect the results. The number of studies included in the present review was relatively small considering the age group, the different language profiles included, and the variety of intervention foci. Further, only eight of the 25 studies (32%) mentioned the risk of bias or how the researchers tried to minimise it. The bias was minimised by blinded scorers (Phillips, 2014), fidelity assessment, having two raters (Roberts & Kaiser, 2015), choosing optimal effect size measure (van Kleeck et al., 2006), “conducting trim and fill” procedure (Roberts & Kaiser, 2011), and attempting to discern the possibility that the small differences in treatment focus across clinicians could have biased the data in some way (Fey et al., 2010). The risk of bias was recognised as sometimes the assessors (Colmar, 2014) as well as caregivers could not remain naïve to the trial arm (Roberts & Kaiser, 2015). Still, the lack of blinding did not always seem to bias outcomes. In the study of Wake et al. (2013), there were beneﬁts in 3 of the 4 directly assessed outcomes, but none of the parent-reported outcomes, and researchers interpreted that the lack of parent blinding did not bias outcomes. The risk of selection bias was also acknowledged (Hagen et al., 2017). The low number of studies reporting on bias limits the confidence one can have in the results. In addition, only 24% of the studies reported information about maintenance, and 40% of the studies about generalisation. This limits the interpretation of the long-term benefits of the interventions. Furthermore, none of the assessment methods in the 25 studies targeted children’s opinions or their experiences related to their skills or coping in everyday life. It is therefore unclear how well children can use their learned skills, and thus, the clinical significance of these interventions remains obscure.

### Clinical implications

The need for further intervention studies is evident. Some implications to clinical practice can be concluded, however. The findings suggest that guiding parents in their communication strategies is one of the possible ways of improving young children’s oral language comprehension. The quality and quantity of the parent–child interaction are known to be associated with language development (Gilkerson et al., 2018; Roberts et al., 2019). The results of this review support the view that oral language comprehension skills of young children can be improved by targeting parents’ communication strategies. Though the severity of the language disorder (Bishop et al., 2017) and intelligence quotient of the child (Davis et al., 2016) impact the speed of learning, in general younger children respond to intervention faster than older children (Jacoby et al., 2002). This further supports the rationale for early interventions.

Further, clinician- or paraprofessional-implemented interventions can also be used to improve children’s oral language comprehension. These interventions can have a positive impact on children’s oral language comprehension by improving receptive vocabulary, receptive morphosyntax, narrative comprehension, and language processing by reducing the burden on verbal working memory. The two articles included in the present review on inferential language do not offer much support for the efficacy of targeting inferential language. A study identified outside the search of this review, however, indicates that targeting inferential comprehension with dialogic reading can have a positive impact on oral language comprehension (Dawes et al., 2019). A meta-analysis on scaffolding narrative skills indicates medium effect size on comprehension (Pesco & Gagné, 2017) conforming targeting narratives as one of the means to aid oral language comprehension.

### Further research

There is an obvious need for oral language comprehension intervention research. Further efficacy research is needed to examine, for example, semantic and phonological methods, Words of Oral Reading and Language Development, dialogic reading, and mental imagery. RCTs of specific therapy techniques followed by systematic reviews are necessary to verify the efficacy of each therapy technique. The effectiveness, that is, how the intervention works in a real-life setting, of the interventions that have robust evidence of their efficacy should also be examined. Maintenance and generalisation of acquired skills should be examined more systematically in future studies. Further, a review of oral language comprehension interventions in school-age children aged 9 years and older as well as adolescents would be of clinical interest. To better understand the qualities of oral language comprehension interventions indicating efficacy, an analysis of the intervention characteristics associated with positive effect sizes is also warranted.

## Conclusions

This review is the first to summarise the findings of oral language comprehension interventions in young children with language disorders and difficulties. Although DLD with language comprehension problems is a lifelong condition, the results of this review indicate that oral language comprehension difficulties can be ameliorated with well-chosen interventions and by collaborating with people in the child’s surroundings. Considering the persistent nature of oral language comprehension difficulties, and the risks that children with oral language comprehension difficulties are exposed to, this information is of clinical importance. Children with oral language comprehension difficulties should be provided with appropriate interventions. This review provides more knowledge about oral language comprehension interventions for clinical settings. The growing possibilities to employ evidence-based practice have the potential to minimise the risks and enhance the future prospects of individuals with difficulties in oral language comprehension.

## Notes

1 In the present review receptive language refers only to oral language comprehension, and thus excludes reading comprehension.

2 The present review is a part of a larger search on oral language comprehension interventions, also including older children and adolescents.
